# Physiological predictors of survival during high-frequency oscillatory ventilation in adults with acute respiratory distress syndrome

**DOI:** 10.1186/cc12550

**Published:** 2013-03-04

**Authors:** Luigi Camporota, Tony Sherry, John Smith, Katie Lei, Angela McLuckie, Richard Beale

**Affiliations:** 1 Division of Asthma, Allergy and Lung Biology, King's College London and Department of Adult Critical Care, Guy's and St Thomas' NHS Foundation Trust, King's Health Partners, St Thomas' Hospital, 1st Floor East Wing, Lambeth Palace Road, London, SE1 7EH, UK

## Abstract

**Introduction:**

Data that provide clinical criteria for the identification of patients likely to respond to high-frequency oscillatory ventilation (HFOV) are scarce. Our aim was to describe physiological predictors of survival during HFOV in adults with severe acute respiratory distress syndrome (ARDS) admitted to a respiratory failure center in the United Kingdom.

**Methods:**

Electronic records of 102 adults treated with HFOV were reviewed retrospectively. We used logistic regression and receiving-operator characteristics curve to test associations with oxygenation and mortality.

**Results:**

Patients had severe ARDS with a mean (SD) Murray's score of 2.98 (0.7). Partial pressure of oxygen in arterial blood to fraction of inspired oxygen (PaO_2_/FiO_2_) ratio and oxygenation index improved only in survivors. The earliest time point at which the two groups differed was at three hours after commencing HFOV. An improvement of >38% in PaO_2_/FiO_2 _occurring at any time within the first 72 hours, was the best predictor of survival at 30 days (area under the curve (AUC) of 0.83, sensitivity 93%, specificity 78% and a positive likelihood ratio (LR) of 4.3). These patients also had a 3.5 fold greater reduction in partial pressure of carbon dioxide in arterial blood (PaCO_2_). Multivariate analysis showed that HFOV was more effective in younger patients, when instituted early, and in patients with milder respiratory acidosis.

**Conclusions:**

HFOV is effective in improving oxygenation in adults with ARDS, particularly when instituted early. Changes in PaO_2_/FiO_2 _during the first three hours of HFOV can identify those patients more likely to survive.

## Introduction

Patients with acute respiratory distress syndrome (ARDS) exhibit a highly inhomogeneous, compliance-dependent distribution of regional ventilation during conventional mechanical ventilation (CMV) [1]. Consequently, CMV can lead to further lung injury through tidal hyperinflation and shear stress injury, even when it is administered according to a 'lung protective strategy' that limits tidal volumes and plateau pressure [2], and employs recruitment maneuvers to maximize the proportion of aerated alveolar tissue [1]. High-frequency oscillatory ventilation (HFOV) can theoretically offer effective lung protective ventilation by delivering very low tidal volumes (1 to 3 mL Kg^-1^) around a fixed mean airway pressure at frequencies of 5 to 12 Hz (lower frequencies are used in adults). At high respiratory frequencies, the short inspiratory time results in a distribution of ventilation which, compared to mechanical ventilation at conventional breathing rates, is more homogeneous and less dependent on the distribution of regional lung compliance [3,4]. This results in the protection of the recruited lung (with greater compliance) from excessive cyclic variations in alveolar pressure. In addition, if the continuous distending pressure (CDP) is optimized following a stepwise recruitment maneuver, the more compliant lung regions are less susceptible to static hyperinflation [3,5,6], thereby reducing lung strain and ventilation-induced inflammation [7,8].

HFOV improves gas exchange and reduces lung injury in animal models of ARDS [9-11] and in human neonatal and pediatric populations [12-15]. In adults the effects of HFOV are largely limited to observational studies [16-24] and two randomized trials [25,26]. Overall these studies show that HFOV might improve gas exchange and survival. However, data that provide clinical criteria for identification of patients likely to benefit from HFOV are scarce [23,24]. Large studies outside North America, utilizing different protocols are lacking, although the results of two multicenter clinical trials in the UK and Canada (OSCAR and OSCILLATE trials) are awaited [16,27,28].

In this study we aim to describe potential physiological predictors of survival during HFOV in adults with severe ARDS admitted to an advanced respiratory failure center in the United Kingdom.

## Materials and methods

### Patients

This was a single center observational study of patients with ARDS admitted to the Adult Intensive Care Service at Guy's and St Thomas' Hospital in London between 1998 and 2002. We included patients who were treated with HFOV because of severe gas exchange impairments while on CMV. Medical records and physiological data before, during and after HFOV were retrieved from our ICU electronic patient record (Intellivue Clinical Information Portfolio, Philips Medical Systems UK Limited). Patients' demographic data, hemodynamic variables, oxygenation and ventilator settings were recorded while on CMV prior to HFOV, during HFOV, prior to discontinuation of HFOV and on recommencement of CMV. Oxygenation index (OI = (FiO_2_CDP 100)/PaO_2_) and partial pressure of oxygen in arterial blood to fraction of inspired oxygen (PaO_2_/FiO_2_) ratio were calculated at the same intervals. Acute Physiology and Chronic Health Evaluation (APACHE II) and Murray lung injury severity score (which combines degree of lung infiltration on chest × ray, lung compliance, PaO_2_/FiO_2 _and positive end expiratory pressure (PEEP)) [29] at admission and on commencement of HFOV were determined (with a blinded and independent radiologist scoring the chest x-ray appearance). Hemodynamic data were obtained using cardiac output monitors PiCCOplus (Pulsion, Munich, Germany, Software version 7.0 non USA), or a LiDCO (LiDCO Ltd, London, UK). This study was considered by the National Research Ethics Service as 'service evaluation' and therefore did not require Research Ethics Committee review [30].

### Ventilator settings and study protocol

All patients were ventilated with pressure-controlled ventilation before starting HFOV, using a lung protective strategy [31,32]. Patients were considered for HFOV if SaO_2 _<88%/PaO_2 _<60 mmHg, FiO_2 _>0.6 and pH <7.2.

HFOV was delivered using an adult high-frequency oscillatory ventilator (3100B, Viasys (CareFusion), Yorba Linda, CA, USA). All patients were initiated onto HFOV using the following settings: a FiO_2 _of 1.0, a frequency of 4 to 6 Hz, inspiratory time of 33%, a bias flow of 30 to 40 Lmin^-1^, a CDP set 3 to 5 cmH_2_O above the CDP during CMV and a Power to obtain transmitted oscillation ('wiggles') up to the level of mid-thigh. The power dial determines the amount of power that drives the oscillator piston to and fro. The Power control is a 10-turn locking dial, electrical potentiometer covering the power range of 0 to 100%. The effect of this control is to change the displacement of the oscillator piston and hence to determine the oscillatory pressure ΔP. The Power setting interacts with the pulmonary artery wedge (Paw) and the conditions existing within the circuit to produce the resultant ΔP [33].

On starting HFOV, patients underwent a standardized slow recruitment maneuver (SRM), which represents the standard of care for patients receiving HFOV in this Institution. The SRM is derived from the maneuver included in the original MOAT Study protocol [34]. The SRM was performed by a stepwise increase in CDP by increments of 3 cmH_2_O every 10 minutes, starting from the CDP on CMV + 3 to 5 cmH_2_O, up to 50 cmH_2_O. SRM was interrupted if the mean arterial pressure fell below 55 mmHg or if desaturations (SpO2 <85%) or arrhythmias occurred. Subsequently, CDP was reduced by 2 cmH_2_O every 5 minutes. Arterial blood gases were taken every 10 minutes (every step during the incremental CDP, phase, and every two steps of the decremental CDP, phase). The 'optimal' CDP was established as the lowest CDP that achieved the most favorable combination between the highest PaO_2 _and/or lowest PaCO_2_, while maintaining the FiO_2 _constant at 1.0.

The protocol for the adjustment of HFOV was published previously [25]. A reduction of CDP was initiated when the FiO_2 _was ≤0.5. Once CDP ≤20 to 22 cmH_2_O was achieved on a FiO_2 _of 0.4, the patients returned to CMV. CMV was restarted in the pressure-control mode with CDP close to the CDP on HFOV, plateau pressures <28 cmH_2_O and PEEP adjusted to a tidal volume of 6 mLKg^-1 ^of predicted body weight.

### Outcome measures

The primary outcome measures were: improvement in PaO_2_/FiO_2 _and OI and identification of physiological variables associated with 30-day survival.

To stratify patients we used an empirical score generated from the available data that was solely designed to give a pragmatic quantification of disease severity and not intended to have diagnostic or prognostic value. The score included PaO_2_/FiO_2_, basal PaCO_2_, respiratory system compliance, minute ventilation and mean airway pressure. This score was used as a dichotomous variable to separate patients with a more severe index of disease (score >50th percentile) from those with a less severe index of disease (score <50th percentile).

### Statistical analysis

Distribution of baseline variables was assessed using the Kolmogorov-Smirnov test. Differences in baseline variables between survivors and non-survivors were compared using the two-tailed t-test or Mann-Withney U test for continuous data, and χ^2 ^or the Fisher test for qualitative data. Differences in physiological variables over time between the two outcome groups were evaluated using repeated-measure analysis of variance (ANOVA). The Friedman test and Dunn's *post-hoc *analysis was performed for non-normally distributed data. Multiple regression analysis and analysis of co-variance (ANCOVA) were used to test the effect of various physiological variables on oxygenation indices. Continuous outcome variables were corrected for confounding variables at baseline. *Post hoc *analyses were performed using Bonferrroni's correction. Variables associated with mortality in an analysis of covariance were entered in a multivariate logistic backward-likelihood ratio regression analysis, to identify predictors of mortality at different end-points. The Hosmer-Lemeshow goodness-of-fit test was used to test the validity of the model. Receiver-operating characteristic (ROC) curves were plotted to determine the best predictor of survival. The value with the best sensitivity, specificity and positive predictive value was selected as the cut-off point to predict survival.

Analyses were performed using SPSS software (version 12; SPSS, Chicago, IL, USA) and MediCalc (Mariakerke, Belgium) for ROC curve analysis. Two-tailed tests for significance were used, and a *P *value less than 0.05 was considered statistically significant.

## Results

We report the results on 102 consecutive ARDS patients who received HFOV. The median (IQR) duration of ARDS prior to HFOV was 48 hours (24 to 120 hours). The median (IQR) duration of CMV prior to HFOV was 45 hours (9 to 138 hours). Table 1 presents the baseline patient demographics, physiological variables and severity scores at study entry. Table 2 summarizes patients' outcome and complications from HFOV.

**Table 1:** Patient characteristics at study entry

Variable	
Patient characteristics	
Patients, number	102
Age, years	50.8 ± 15.9
Gender, % male	67.6
Actual body weight, Kg	75.7 ± 21.24
APACHEII prior HFOV	24.1 ±8.0
Murray Score	2.98 ± 0.7
Duration ARDS prior HFOV, hours median (IQR)	48 (24 to 120)
Duration CMV prior HFOV, hours median (IQR)	45 (9 to 138)
Gas exchange on CMV pre-HFOV	
PaO_2_, mmHg	74.2 ± 21
PaCO_2_, mmHg	57.6 ± 18.7
PaO_2_/FiO_2_, mmHg	93.8 ± 38.3
SaO_2_, %	88.6 ± 11.99
OI, cmH_2_· mmHg^-1^	27 ± 13.4
pH	7.26 ± 0.14
Respiratory variables on CMV pre-HFOV	
PIP, cmH_2_O	32.1 ± 5.67
PEEP, cmH_2_O	12.4 ± 3.7
CDP, cmH_2_O	21.6 ± 4.97
Compliance	26.4(19.9 to 36.3)
Minute ventilation, L^.^min^-1^	10.4 (8.5 to 12.6)
Respiratory rate	20.9 ± 5.1
Tidal volume on CMV, mL	551 (421.7 to 620)
Hemodynamics on CMV pre-HFOV	
PAWP, cmH_2_O	16.8 ± 7.1
Cardiac Output, L·min^-1^	5.95 ± 1.7
Etiology of ARDS	
Sepsis	69
Pulmonary infection	13
Trauma	3
Aspiration	2
Pancreatitis	2
Drug-induced	1
Other	12

Data are presented as absolute number, % or mean ± standard deviation (SD) unless otherwise indicated. CDP, continuous distending pressure; CMV, conventional mechanical ventilation; CO, cardiac output; FiO_2, _fraction of inspired oxygen; HFOV, high-frequency oscillatory ventilation; PAWP, pulmonary artery wedge pressure; PEEP, positive end-expiratory pressure; PIP, peak inspiratory pressure; OI, oxygenation index (OI = (FiO_2_•CDP•100)/PaO_2_).

**Table 2:** Patient outcomes and complications

Variable	
Airleak	
Air Leak before HFOV	22/102 (21.6 %)
Persistent air Leak during HFOV	5/22 (22.7 %)
New air leak during HFOV	2/80 (2.5 %)
Co-treatment (Some patients received more than one co-treatment)	
Nitric oxide	21/102 (20.6%)
MARS	1/102 (0.98%)
Steroids	19/102 (18.6%)
Prone position	20/102 (19.6%)
rhAPC	3/102 (2.9%)
None	56/102 (54.9%)
Mortality	
Post HFOV	49/102 (48%)
ICU discharge	62/102 (60.8%)
30-day	57/102 (55.9%)
Cause of death at 30-days	
Withdrawal of treatment	22/57 (38.6%)
>2 organ failure	20/57 (35.1%)
Cardiac arrhythmia	6/57 (10.5%)
Refractory hypoxia	4/57 (7%)
Sepsis	3/57 (5.3%)
Profound hypotension	2/57 (3.5%)

HFOV, high-frequency oscillatory ventilation; MARS, Molecular Adsorbents Recirculation System; rhAPC, recombinant human Activated Protein C (Drotrecogin alfa activated, Xigris).

Overall, HFOV was well tolerated with low incidence of new or worsening pneumothoraces, pneumomediastinum or subcutaneous emphysema (2%) and hemodynamic compromise. Two patients (1.96%) suffered profound hypotension during HFOV.

### Effects of HFOV on gas exchange

**Figure 1:**
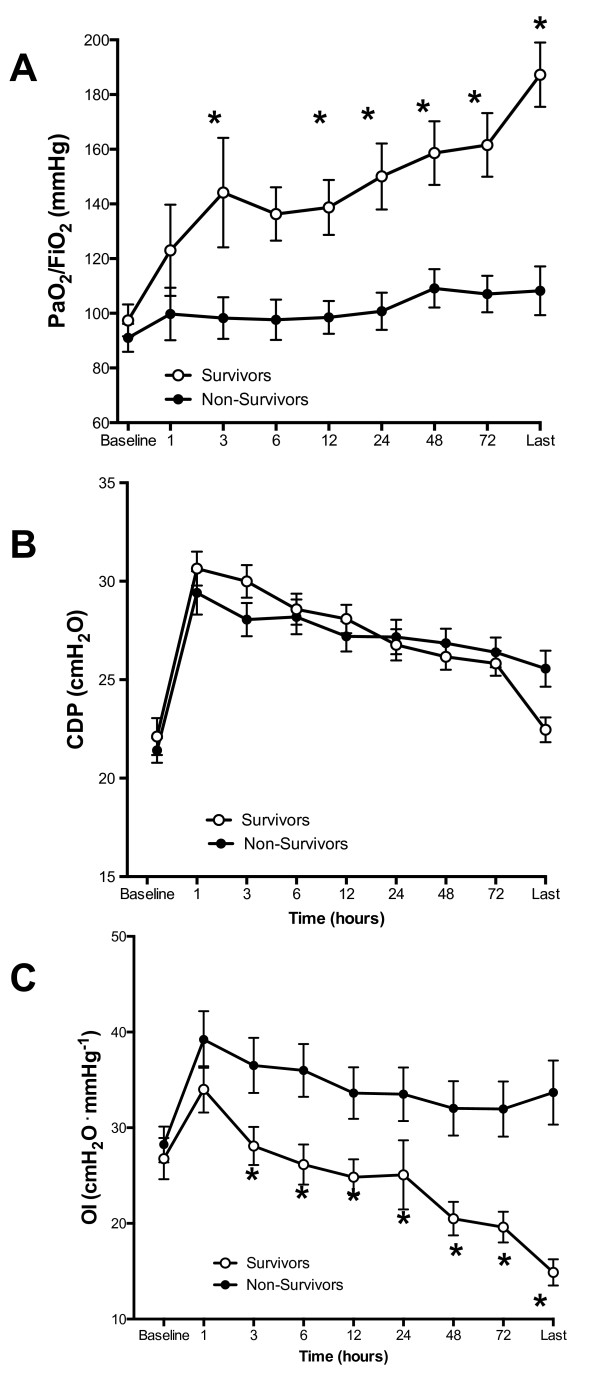
**Differences in PaO_2_/FiO_2 _ratio, CDP and OI in survivors (open circles) and non-survivors (filled circles)**. X-axis indicates time (hours) on HFOV. Baseline is the time on CMV immediately preceding the change to HFOV. 'Last' is the last measurement on HFOV prior to returning to CMV. **A**: Differences PaO_2_/FiO_2 _ratio over time between survivors (open circles) versus non-survivors (filled circles). **B**: Trends of change in CDP between survivors and non-survivors. Data are displayed as mean and error bars represent SEM at each time-point. The number of patients (survivors - S; and non-survivors -NS) at different time- points were: at baseline (S, *n *= 45; NS, *n *= 57); 12 hours (S, *n *= 44; NS, *n *= 38); 24 hours (S, *n *= 39; NS, *n *= 28); 48 hours (S, *n *= 31; NS, *n *= 24); 72 hours(S, *n *= 25; NS, *n *= 19). * = *P *<0.01 - comparisons at each timepoint *versus *baseline. CDP, continuous distending pressure; CMV, conventional mechanical ventilation; FiO_2, _fraction of inspired oxygen; HFOV, high-frequency oscillatory ventilation; SEM, standard error of the mean.

During the first 72 hours of HFOV, PaO_2_/FiO_2 _improved significantly from baseline only in survivors (Figure 1a). The earliest time-point at which PaO_2_/FiO_2 _was statistically different from baseline in the survivor group was at three hours of HFOV (*P *<0.05) (Figure 1a). The improvement in PaO_2_/FiO_2 _was not determined by the level of CDP (Figure 1b) with mean CDP ± SD of 33.9 ± 5.4 cmH_2_O versus 32.0 ± 7.05 cmH_2_O (*P *= 0.08), respectively, for survivors and non-survivors. The change in PaO_2_/FiO_2 _remained significantly different (*P *= 0.03) between the two outcome groups after adjusting for baseline confounding factors such as age, PaO_2_/FiO_2 _and CDP. The independence from CDP during HFOV was further demonstrated by the divergence of OI between the two groups and the fact that OI improved significantly over the first 72 hours only in survivors (Figure 1c). Analysis of ROC curves identified an improvement of 38% in PaO_2_/FiO_2 _and an improvement of >22% in the OI during the first 72 hours of HFOV as the criteria with the best positive predictive values for survival, with respective sensitivities of 93.3% and 87%, specificities of 78.3% and 78.0% and positive likelihood ratios of 4.29 and 3.96. Change in PaO_2_/FiO_2 _was a better indicator of survival compared with the change in OI, with an area under the ROC curve of 0.83 (95% CI, 0.71 to 0.92) versus 0.69 (95 % CI 0.55 to 0.8) (*P *= 0.039, pair-wise comparison of ROC curves).

Multivariate logistic regression analysis identified the following four independent predictive factors of mortality at 30 days: 1) days with ARDS prior to HFOV (OR 1.5, 95%CI 1.08 to 1.92; *P *= 0.01); 2) improvement in PaO_2_/FiO_2 _in the first 72 hours (OR 0.8, 95 % CI 0.77 to 0.9; *P *<0.001), 3) age (OR 1.1, 95 % CI 1.02 to 1.14; *P *= 0.03); and 4) pre-HFOV pH (OR 0.8, 95 % CI 0.7 to 0.9; *P *= 0.004). The change in PaO_2_/FiO_2 _and OI after three hours of HFOV was the earliest time point to predict outcome.

There was an interaction between change in PaO_2_/FiO_2 _and the etiology of ARDS, with a greater change in PaO_2_/FiO_2 _for patients with extra-pulmonary ARDS, independent of baseline PaO_2_/FiO_2_, which may reflect the different degree of lung recruitability.

### Effects of HFOV on PaCO_2_

**Figure 2:**
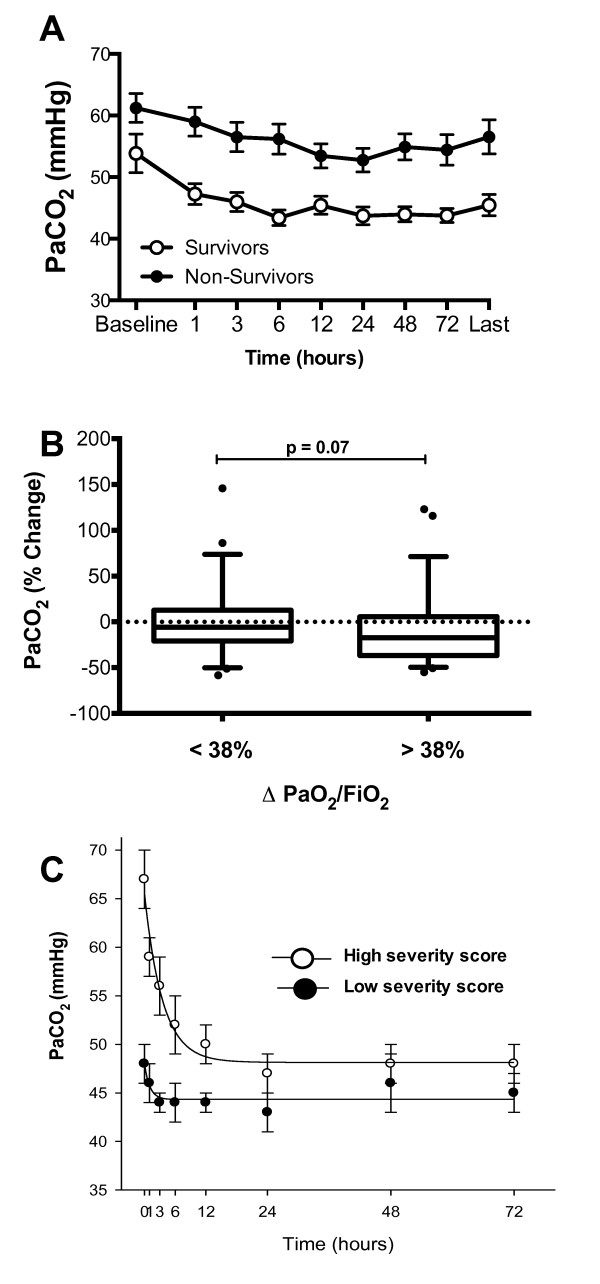
**Changes in PaCO2 based on survival status, response to HFOV and disease severity**. **A**: Differences in PaCO_2 _in survivors (open circles) and non-survivors (filled circles) over 72 hours of HFOV. X-axis indicates time (hours) on HFOV. Time 0 is the time on CMV immediately preceding the change to HFOV. 'Last' is the last measurement on HFOV prior to returning to CMV. There was no difference in the trend of change in PaCO_2 _between the two outcome groups. Data are displayed as mean and error bars represent SEM at each time-point. **B**: Patients were subdivided into two groups (responders and non-responders) based on the analysis of the ROC curve. They were considered responders if their PaO_2_/FiO_2 _ratio improved >38% from baseline. Responders show a trend towards better CO_2 _clearance (*P *= 0.07). Data are displayed as mean and error bars represent SEM. **C**: Patients were subdivided into two groups based on disease severity. Patients with more severe disease (open circles) have a greater clearance in PaCO_2_. The number of patients (Survivors - S; and non-survivors -NS) at different time- points after initiating HFOV were: at baseline (S, *n *= 45; NS, *n *= 57); 12 hours (S, *n *= 44; NS, *n *= 38); 24 hours (S, *n *= 39; NS, *n *= 28); 48 hours (S, *n *= 31; NS, *n *= 24); 72 hours (S, *n *= 25; NS, *n *= 19). CMV, conventional mechanical ventilation; FiO_2, _fraction of inspired oxygen; HFOV, high-frequency oscillatory ventilation; PaCO_2_, partial pressure of carbon dioxide in arterial blood; ROC, receiver-operating curve; SEM, standard error of the mean.

Survivors had a lower baseline PaCO_2 _with a median (IQR) of 47 mmHg (38.6 to 62.2 mmHg) versus 58 mmHg (47.5 to 72.3 mmHg) (*P *= 0.008) and lower PaCO_2 _throughout HFOV treatment (*P *<0.001) and on return to CMV (Table 3). Overall, PaCO_2 _decreased significantly throughout the duration of HFOV (*P *= 0.001, repeated measure ANOVA) and, at each time-point, PaCO_2 _was significantly lower than baseline in both groups (*P *<0.001) (Figure 2a).

**Table 3:** Patient characteristics at 30 days: comparison of survivors versus non-survivors

Variable	Survivors (number = 45)		Non-survivors (number = 57)		
	**Mean**	**SD**	**Mean**	**SD**	***P *value**

Demographics					
Age, years	45.7	16.2	54.9	14.6	0.003
Weight, Kg	75.8	22.7	75.6	20.2	ns
APACHE II (prior to HFOV)	22.9	6.52	24.98	8.96	ns
Murray Score	3.0	0.62	2.9	0.77	ns
Duration ARDS prior to HFOV, days	2.9	3.6	4.5	4.7	0.015
Duration CMV prior to HFOV, hours	88.9	131.9	90.4	105.9	ns
Gas exchange indices					
PaO_2_/ FiO_2 _prior to HFOV, mmHg	96.8	38.8	90.8	38.3	ns
PaO_2_/ FiO_2 _return to CMV, mmHg	211.5	96.0	129.0	70.5	0.001
Δ PaO_2 _/ FiO_2_	111.0	89.3	26.7	63.0	<0.01
PaCO_2 _prior to HFOV, mmHg	54.0	20.8	60.8	17.0	ns
PaCO_2 _return to CMV, mmHg	50.3	17.3	81.8	97.4	0.049
OI prior HFOV, cmH_2_O· mmHg^-1^	26.1	13.4	27.6	13.4	ns
OI return to CMV, cmH_2_O· mmHg^-1^	10.1	6.6	21.8	19.1	0.01
Max PaCO_2_, mmHg	55.5	10.6	66.7	18.8	<0.01
Min PaCO_2_, mmHg	36.0	6.7	47.9	16.0	<0.01
pH prior to HFOV	7.3	0.14	7.23	0.12	<0.01
pH return to CMV	7.4	0.1	7.27	0.1	<0.01
Hemodynamic indices					
MAP prior to HFOV, mmHg	76.3	15.4	75.1	12.9	ns
MAP return to CMV, mmHg	82.9	14.6	77.0	20.9	ns
CO prior to HFOV, L · min^-1^	6.1	2.0	5.8	1.5	ns
CO return to CMV, L ·min^-1^	5.9	1.3	6.1	0.5	ns
Ventilator Indices					ns
Vt prior to HFOV, mL ·Kg^-1^	7.4	2.4	6.9	2.2	ns
Vt return to CMV, mL ·Kg^-1^	6.9	2.1	6.8	1.9	ns
PIP prior to HFOV, cmH_2_O	31.8	6.2	32.3	5.2	ns
PIP return to CMV, cmH_2_O	28.5	5.2	30.3	5.98	ns
PEEP prior to HFOV, cmH_2_O	12.9	4.0	12.1	3.4	ns
PEEP return to CMV, cmH_2_O	10.5	2.7	10.8	3.3	ns
CDP prior to HFOV, cmH_2_O	21.8	5.7	21.4	4.3	ns
CDP return to CMV, cmH_2_O	17.5	4.5	20.7	5.9	0.02
Δ CDP, cmH_2_O	-3.9	6.5	-0.57	4.6	0.03

CDP, continuous distending pressure; CMV, conventional mechanical ventilation; CO, cardiac output; FiO_2_, fraction of inspired oxygen; HFOV, high-frequency oscillatory ventilation; OI,Oxygenation Index (OI = (FiO_2_•CDP•100)/PaO_2_); PAWP, pulmonary artery wedge pressure; PEEP, positive end-expiratory pressure; PIP, peak inspiratory pressure; Vt, tidal volume; = change post-HFOV to pre-HFOV.

**Figure 3:**
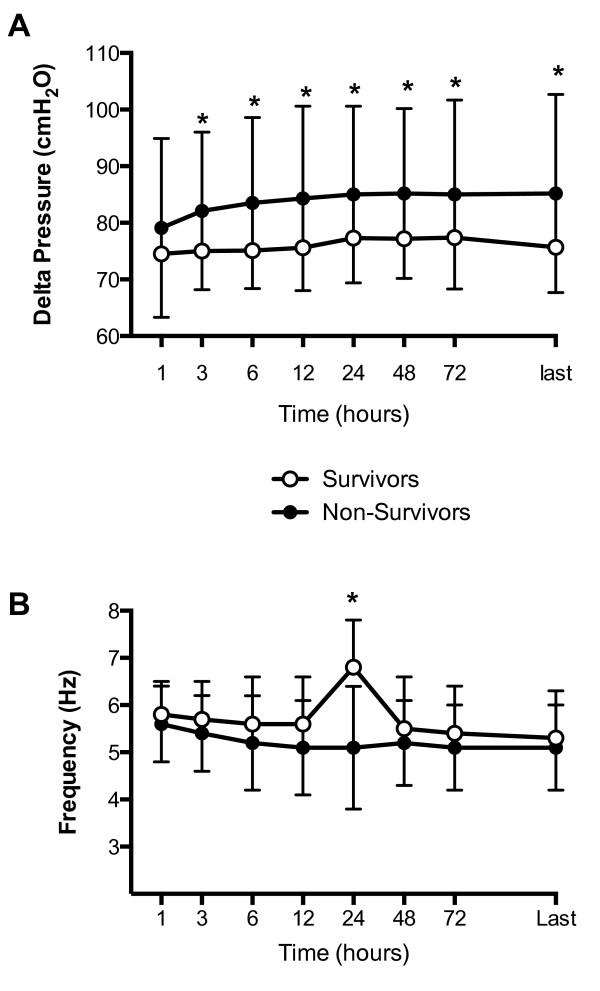
**Differences in HFOV settings over time between survivors and non-survivors**. **A**: Differences in Delta pressure (ΔP) in survivors (open circles) and non-survivors (filled circles) over 72 hours of HFOV. There was a significant difference in the ΔP between the two outcome groups. Data are displayed as mean and error bars represent SD at each time-point. * = *P *<0.01 between the two groups. **B**: Differences in frequency in survivors (open circles) and non-survivors (filled circles) over 72 hours of HFOV. There was a significant difference in the frequency between the two outcome groups at 24 hours. Data are displayed as mean and error bars represent SD at each time-point. * = *P *<0.01 between the two groups. HFOV, high-frequency oscillatory ventilation.

There was a trend towards greater reduction in PaCO_2 _in 'responders' as defined on the ROC curve by an increase in PaO_2_/FiO_2 _ratio of >38 % compared to 'non-responders' (increase in PaO_2_/FiO_2 _ratio of <38 %), with a median per cent change (IQR) of -17.4 % (-33.4 to 5.48) versus -4.9 % (-19.8 to 11.9) (*P *= 0.07) (Figure 2b). Furthermore, patients with a worse empirical disease severity score showed a more rapid clearance of PaCO_2 _during the first 12 hours of HFOV (Figure 2c) despite similar settings of frequency, amplitude and power (Figure 3). The absolute PaCO_2 _remained higher in the more severe group. This result may suggest that patients with more severe disease have a greater proportion of recruitable lung and an increase in alveolar ventilation following HFOV. Overall, patients with lower respiratory system compliance had a trend towards a greater change in PaCO_2 _post-SRM (-20.5% versus -2.4 %; *P *= 0.08), and there was a correlation between change in PaCO_2 _post-SRM and change in compliance post-HFOV (r^2 ^= 0.6; *P *= 0.04).

## Discussion

This study aimed to identify potential predictors of survival in patients with severe ARDS who received HFOV after failing lung-protective CMV. The key results of our study are that: 1) an early improvement in PaO_2_/FiO_2 _ratio is a predictor of survival at 30 days; 2) patients with more severe disease and lower respiratory system compliance pre-HFOV show greater CO_2 _clearance; and 3) if there is no improvement in gas exchange within three hours, patients can be considered to have failed HFOV and perhaps should be considered for alternative treatment (for example, extracorporeal support).

Despite theoretical beneficial effects on minimizing lung injury and improving gas exchange, HFOV is not widely utilized because of the lack of evidence supporting a clear survival benefit over CMV [25,26,34]. Furthermore, available clinical trials do not help the clinician decide when to consider HFOV and, importantly, how long HFOV should be continued to enhance patient survival. Our study has a similar scope to the series reported by Adhikari *et al. *[23]; however, there are important methodological differences in the HFOV protocols and the type of recruitment maneuver (that is, a slow stepwise maneuver in our study versus a sustained inflation in Adhikari *et al*.) used in the two studies. Furthermore, recruitment maneuvers were performed in all patients in our case series, whereas in the study by Adhikari *et al *only 49.5% of the patients received a recruitment maneuver. The rationale of the stepwise recruitment we used in this study was similar to the stepwise recruitment used in neonates [35], in that it allowed for setting of the optimal CDP but it differed in two aspects. First, we used a fixed FiO_2 _of 1.0 and response to the recruitment was assessed as changes in PaO_2_. Second, in order to allow time for equilibration of PaO_2 _[36] and to minimize hemodynamic instability, our protocol required longer times between changes in CDP (ten minutes during the incremental phase and five minutes during the decremental phase) compared to the recruitment used in neonates [35]. It is possible that the slower and early recruitment, as performed in this study, can explain the early identification of responders to HFOV.

Although our study is not a randomized comparative study, we believe it identifies clinically important predictors of clinical outcome within the first few hours of initiation of HFOV, possibly in response to the initial SRM. Our study population included, as might be expected for a rescue study, patients with more severe ARDS than in the MOAT trial where patients received HFOV as a primary ventilation mode [26] and similar to that of the EMOAT trial [25] and the recent case series[23].

In contrast to other published reports [17,18], in our study gas exchange variables (PaO_2_/FiO_2_, and OI) improved significantly only in survivors, and change in PaO_2_/FiO_2 _remained significantly different between the two outcome groups after adjusting for baseline confounding factors. Although the CDP on HFOV was higher than the CDP on CMV, there was no difference in CDP between survivors and non-survivors. Despite similar levels of CDP, the response to HFOV within the first three hours could identify patients with a favorable outcome based on PaO_2_/FiO_2 _and OI.

An important factor for the response to HFOV may be played by the proportion of patients with pulmonary versus extra-pulmonary ARDS. Indeed, in this study we show that the largest change in PaO_2_/FiO_2 _post HFOV was seen for extra-pulmonary ARDS whereas little difference in PaO_2_/FiO_2 _was seen in pulmonary ARDS. This is consistent with the findings reported by Pachl *et al*. that show that oxygenation and recruitment during HFOV are more pronounced in patients with extra-pulmonary ARDS [37]. However, Pachl *et al*. studied changes in oxygenation under normocapnic conditions; therefore, no data on the different behavior of PaCO_2 _in the two types of ARDS are available for comparison with our data. The other important finding of our study is the effect of HFOV on PaCO_2_. In our study, in contrast to other reports [19,26], the PaCO_2 _decreased significantly throughout the duration of HFOV in parallel to an increase in PaO_2_/FiO_2 _despite similar settings of frequency, power and amplitude. We found that in survivors there was both an increase in PaO_2_/FiO_2 _and a decrease in PaCO_2. _In addition, patients who had at least a 38% increase in their PaO_2/_/FiO_2 _(as identified by the ROC curve), also showed greater reductions in PaCO_2 _allowing for a reduction in delta pressure. Patients with a greater disease severity (higher PaCO_2, _lower compliance and worse gas exchange), showed a higher rate of clearance in PaCO_2 _during the first six hours of HFOV. These changes in physiological variables have been described in patients with severe ARDS and higher potential for lung recruitment [38].

The increase in intra-thoracic pressure generated during a SRM could have caused a reduction in cardiac output and pulmonary blood flow, leading to a decrease in venous admixture and to an apparent improvement in PaO_2_/FiO_2 _in the absence of true alveolar recruitment [39,40]. However, this mechanism seems less likely as an explanation for the changes seen in our study, as the cardiac output and oxygen delivery were unchanged following the SRM, and therefore the combined improvement in PaO_2_/FiO_2 _and PaCO_2 _leads us to speculate that HFOV facilitated lung recruitment in a manner similar to that described for patients responding to prone positioning [41].

The 30-day mortality in this study was 56%, comparable to the mortality rate reported in other uncontrolled studies (61.7% [23,42], 66% [18], 53% [17]) but higher than the studies using HFOV as primary intervention (43% [25] and 37% [26]) and studies of trauma patients [16]. Multivariate analysis shows that changes in PaO_2_/FiO_2, _age, days with ARDS prior to HFOV and baseline pH are independent predictive factors of mortality. Lung injury is positively associated with duration of mechanical ventilation in both animal and human studies: increased lung injury is associated with reduced likelihood of pulmonary recruitment

## Conclusions

In conclusion, this study shows that HFOV is effective in improving oxygenation in some adults with ARDS, particularly when instituted early. This study also shows that changes in PaO_2_/FiO_2 _are sensitive criteria to predict survival and the change in PaCO_2 _may identify patients with a greater proportion of recruitable lung more likely to benefit from HFOV. Patients who do not show improvement in PaO_2_/FiO_2 _ratio and oxygenation index within six hours on commencing HFOV should be considered for extracorporeal support. These data are of potential value in aiding decision making.

Further randomized controlled trials powered to detect a difference in survival between HFOV strategies are expected. Interpretation of these comparisons will also need to take into consideration the number, the duration, and the type of recruitment maneuvers carried out during HFOV and CMV (for example, slow stepwise RMs, as employed in this study versus traditional RMs, with continuous positive airway pressure (CPAP) of 40 cmH_2_O for 40 seconds). The identification of patients likely to benefit from HFOV and the identification of physiological variables associated with the potential for lung recruitment will prove essential to ensure the best use of HFOV in adults with ARDS.

## Key messages

• Changes in PaO_2_/FiO_2 _early during HFOV are sensitive criteria to predict survival.

• Patients who do not show improvement in the PaO_2_/FiO_2 _ratio and OI within three hours should be considered for alternative treatment (for example, extracorporeal support).

• The identification of patients likely to benefit from HFOV and the identification of physiological variables associated with the potential for lung recruitment will prove essential to ensure the best use of HFOV in adults with ARDS.

## Abbreviations

ANOVA: analysis of variance; APACHE II: Acute Physiology And Chronic Health Evaluation; ARDS: Acute Respiratory Distress Syndrome; CDP: continuous distending pressure; CMV: conventional mechanical ventilation; CPAP: continuous positive airway pressure; CO: cardiac output; ΔP: delta pressure; FiO_2_: fraction of inspired oxygen; HFOV: high frequency oscillatory ventilation; LR: likelihood ratio; MAP: mean arterial pressure; MARS: Molecular Adsorbents Recirculation System; OI: oxygenation index; PaO_2_: partial pressure of oxygen In arterial blood; PaCO_2_: partial pressure of carbon dioxide in arterial blood; PAWP: pulmonary artery wedge pressure; PEEP: positive end expiratory pressure; PIP: peak inspiratory pressure; rhAPC: recombinant human activated protein C (Drotrecogin Alfa Activated); RM: recruitment maneuver; ROC: receiver-operating characteristic; SRM: slow recruitment maneuver.

## Competing interests

The authors declare that they have no competing interests.

## Authors' contributions

LC designed the study, performed the statistical analysis and drafted the manuscript. TS participated in data collection. JS participated in the design of the study, data collection, and data analysis. KL participated in the data collection and analysis. AM participated in the design of the study, data analysis and critical revisions of the draft. RB participated in the design of the study, data analysis and critical revisions of the draft. All authors read and approved the final manuscript.
